# Barriers to the implementation of checklists in the ICU: a survey on the perceptions of 314 Brazilian critical care nurses and physicians

**DOI:** 10.1186/cc14596

**Published:** 2015-03-16

**Authors:** J Salluh, W Viana, F Machado, A Cavalvanti, F Bozza, M Soares

**Affiliations:** 1 IDOR, Rio de Janeiro, Brazil; 2 Hospital Copa D'OR, Rio de Janeiro, Brazil; 3 UNIFESP, São Paulo, Brazil; 4 Hcor, São Paulo, Brazil

## Introduction

Checklists have been used increasingly in the ICU aiming at mitigating avoidable adverse events, but their content is variable and little is known about barriers to their full implementation. This survey reported practices on the use of checklists and perceptions regarding barriers on its implementation.

## Methods

A web-based survey was conducted among a convenience sample of Brazilian ICU nurses and physicians between August and October 2014. Standard descriptive statistics were used.

## Results

A total of 314 professionals, 104 nurses (33.1%) and 210 physicians (66.9%) responded. The majority (82.4%) had more than 5 years of experience in intensive care. Checklists were applied every day, including weekends, in only 49.8% (*n *= 227). When we compared the barriers perceived by those working in smaller versus larger ICUs (<10 beds vs. >20 beds), the absence of a more comprehensive checklist content (93.6% vs. 81.9%, *P *= 0.026), the absence of specialized software or app (80.8% vs. 63.8%, *P *= 0.014), low availability of mobile devices (87.2% vs. 72.4%, *P *= 0.019), Internet unavailability (83.3% vs. 67.6%, *P *= 0.017), and the limited number of computers (88.5% vs. 76.2%, *P *= 0.0366) were the most often barriers to implementation. Checklists were applied with similar frequencies (<3 times a week, three to five times a week, and every day, including on weekends, *P >*0.05) regardless of the ICU size. When the type of tool used (paper vs. electronic) was considered, the main barrier highlighted was the lack of 100% Internet availability in the ICU (64.8% vs. 100%, *P *= 0.009). Users of paper form had higher demands for more comprehensive checklist content (84.3% vs. 63.6%, respectively, *P *= 0.037) and experienced more barriers to team adherence (98.1% vs. 86.4%, respectively, *P *= 0.034) as compared with those using specialized software.

## Conclusion

Although checklists are recognized as valuable tools for the adherence to best practice in the ICU, it is difficult to ensure the uniformity of their daily use. Resource limitations in smaller ICUs and the absence of comprehensive digital tools, mobile devices and Internet availability preclude full compliance at the bedside.

